# 1-(3,4-Dichloro­benz­yl)pyridinium bis­(2-sulfanyl­idene-1,3-dithiole-4,5-dithiol­ato-κ^2^ *S*,*S*′)nickelate(III)

**DOI:** 10.1107/S160053681104267X

**Published:** 2011-10-22

**Authors:** Guang-Xiang Liu

**Affiliations:** a School of Biochemical and Environmental Engineering, Nanjing Xiaozhuang University, Nanjing 211171, People’s Republic of China

## Abstract

The title compound, (C_12_H_10_Cl_2_N)[Ni(C_3_S_5_)_2_], is an ion-pair complex consisting of 1-(3,4-dichloro­benz­yl)pyridinium cations and [Ni(dmit)_2_] anions (dmit = 2-sulfanyl­idene-1,3-dithiole-4,5-dithiol­ate). In the anion, the Ni^III^ ion exhibits a square-planar coordination involving four S atoms from two dmit ligands. In the crystal, weak S⋯S [3.368 (2) and 3.482 (3) Å], Ni⋯S [3.680 (2) Å] and Cl⋯S [3.491 (2) Å] inter­actions and C—H⋯S hydrogen bonds lead to a three-dimensional supra­molecular network.

## Related literature

For general background to the network topologies and applications of bis­(dithiol­ate)–metal complexes, see: Cassoux (1999 ▶). For the synthesis, structures and properties of related complexes containing dmit ligands, see: Akutagawa & Nakamura (2000 ▶); Liu *et al.* (2010 ▶); Li *et al.* (2006 ▶); Zang *et al.* (2006 ▶, 2009 ▶). For the synthesis of a starting material, see: Wang *et al.* (1998 ▶).
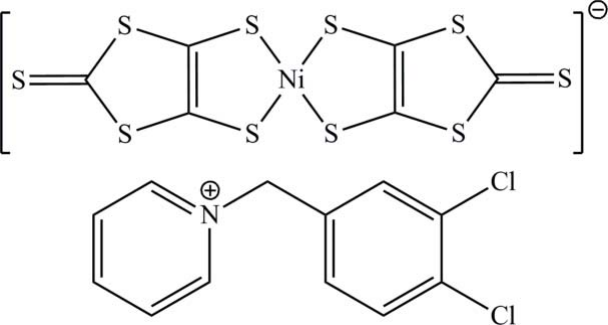


## Experimental

### 

#### Crystal data

(C_12_H_10_Cl_2_N)[Ni(C_3_S_5_)_2_]
*M* *_r_* = 690.48
Triclinic, 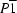

*a* = 9.3711 (11) Å
*b* = 11.7210 (14) Å
*c* = 11.9640 (14) Å
α = 82.814 (1)°
β = 88.854 (1)°
γ = 76.644 (1)°
*V* = 1268.5 (3) Å^3^
*Z* = 2
Mo *K*α radiation
μ = 1.81 mm^−1^
*T* = 293 K
0.22 × 0.20 × 0.16 mm

#### Data collection

Bruker SMART APEX CCD area-detector diffractometer
Absorption correction: multi-scan (*SADABS*; Bruker, 2000 ▶) *T*_min_ = 0.692, *T*_max_ = 0.761
9520 measured reflections
4692 independent reflections
4083 reflections with *I* > 2σ(*I*)
*R*_int_ = 0.028

#### Refinement

*R*[*F* ^2^ > 2σ(*F* ^2^)] = 0.030
*wR*(*F* ^2^) = 0.082
*S* = 1.04
4692 reflections
290 parameters
H-atom parameters constrained
Δρ_max_ = 0.38 e Å^−3^
Δρ_min_ = −0.34 e Å^−3^

### 

Data collection: *SMART* (Bruker, 2000 ▶); cell refinement: *SAINT* (Bruker, 2000 ▶); data reduction: *SAINT*; program(s) used to solve structure: *SHELXS97* (Sheldrick, 2008 ▶); program(s) used to refine structure: *SHELXL97* (Sheldrick, 2008 ▶); molecular graphics: *SHELXTL* (Sheldrick, 2008 ▶); software used to prepare material for publication: *SHELXTL*.

## Supplementary Material

Crystal structure: contains datablock(s) I, global. DOI: 10.1107/S160053681104267X/rz2651sup1.cif

Structure factors: contains datablock(s) I. DOI: 10.1107/S160053681104267X/rz2651Isup2.hkl

Additional supplementary materials:  crystallographic information; 3D view; checkCIF report

## Figures and Tables

**Table 1:** Hydrogen-bond geometry (Å, °)

*D*—H⋯*A*	*D*—H	H⋯*A*	*D*⋯*A*	*D*—H⋯*A*
C14—H14⋯S10^i^	0.93	2.82	3.622 (3)	145
C18—H18⋯S1^ii^	0.93	2.79	3.708 (3)	168

Symmetry codes: (i) 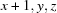; (ii) 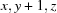.

**Fig. 1.**
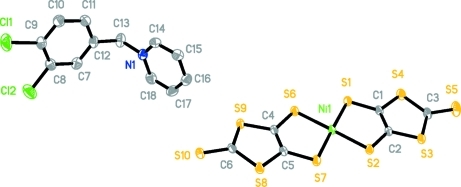
The cation and anion in [DiClPy][Ni(dmit)~2~], showing thermal ellipsoids drawn at the 30% probability level. Hydrogen atoms have been omitted for clarity.

**Fig. 2.**
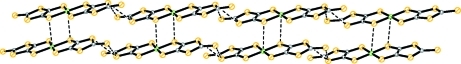
The one-dimensional chain structure of [Ni(dmit)~2~]- anions through S···S and Ni···S contacts. Dashed lines indicate weak interactions.

**Fig. 3.**
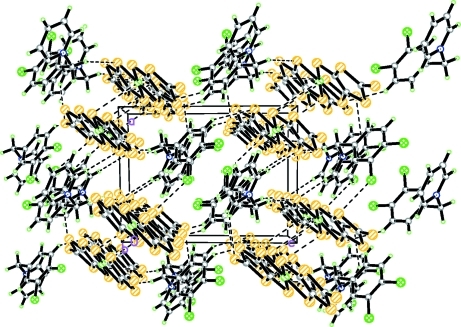
Packing of [DiClPy][Ni(dmit)~2~] viewed along the b axis.

## supplementary crystallographic information

### Comment

Extensive research has been focused on the synthesis and characterization of bis(dithiolate)-metal complexes and their analogues, due to their properties and potential applications as conducting, magnetic and non-linear optical (NLO) materials (Cassoux, 1999). 2-Thioxo-1,3-dithiole-4,5-dithiolate (dmit) metal complexes are in fact excellent building blocks employed for the construction of molecular magnetic materials (Li *et al.*, 2006; Liu *et al.*, 2010; Zang *et al.*, 2006, 2009) apart from their well known electric conductivity as molecular conductors (Akutagawa & Nakamura, 2000). Herein the crystal structure of the title compound, a new ion-pair complex, is reported.

The title compound comprises [Ni(dmit)_2_]^-^ anions and 1-(3,4-dichlorobenzyl)pyridinium cations (Fig. 1). The Ni ion adopts a square-planar geometry coordinated by four S atoms from two dmit ligands, with Ni—S bond lengths ranging from 2.1518 (7) to 2.1714 (7) Å. The [Ni(dmit)_2_]^-^ anions are in a parallel arrangement, with S···S interactions ranging from 3.474 (3) to 3.547 (3) Å. Two neighbouring anions are parallel in a face-to-face fashion with the shortest Ni···S distance of 3.680 (2) Å (Ni1—S2^i^) [symmetry code: (i) -*x*, -*y*, -*z*], indicating the existence of the Ni···S interactions. Adjacent [Ni(dmit)_2_]^-^ anions are associated together through such Ni···S interactions resulting in a dimer. The dimers are linked together through S9···S3^ii^ and S9···S5^ii^ [symmetry code: (ii) *x*, 1 + *y*, *z*] interactions forming a one-dimensional chain structure, as depicted in Fig. 2. The (C_12_H_10_Cl_2_N)^+^ cation has a Λ-shaped conformation, and the dihedral angles formed by the C12/C13/N1 plane with the benzene and pyridinium rings are 85.29 (2) and 77.84 (2)°, respectively. Cations and the anions are linked by S···Cl interactions and C—H···S hydrogen bonds to generate a three-dimensional supramolecular structure (Fig. 3).

### Experimental

4,5-Di(thiobenzoyl)-1,3-dithiole-2-thione (812 mg, 2 mmol; Wang *et al.*, 1998) was suspended in methanol (10 ml). Sodium methoxide in methanol (prepared form 184 mg of sodium in 10 ml of methanol) was added to the above mixture under argon atmosphere at room temperature from 30 min to give a dark red solution. To this solution, NiCl~2~.6H~2Õ (238 mg, 1 mmol) was added. After 30 min, a solution of I~2~ (127 mg, 1 mmol) and NaI (150 mg, 1 mmol) in methanol (20 ml) was added (the monoanionic [Ni(dmit)~2~]^-^ are obtained from the dianionic [Ni(dmit)~2~]^2-^ by I~3~^-^ oxidation). After another 10 min, a solution of 1-(3,4-dichlorobenzyl)pyridinium bromide [(DiClPy)Br] (317 mg, 1 mmol) in methanol (20 ml) was added to the reaction mixture. The solution was stirred for 30 min and cooled in a refrigerator overnight. The resultant dark green crystalline solid was collected by filtration, and purified by recrystallization using a mixed solution of acetonitrile and benzene (1:1 *v*/*v*).

### Refinement

H atoms were positioned geometrically, with C—H = 0.93 and 0.97 Å for aromatic and methylene H atoms, respectively, and constrained to ride on their parent atoms, with *U*_iso_(H) = 1.2 *U*_eq_(C).

### Crystal data

**Table:**

(C_12_H_10_Cl_2_N)[Ni(C_3_S_5_)_2_]	*Z* = 2
*M**_r_* = 690.48	*F*(000) = 694
Triclinic, *P*1	*D*_x_ = 1.808 Mg m^−^^3^
Hall symbol: -P 1	Mo *K*α radiation, λ = 0.71073 Å
*a* = 9.3711 (11) Å	Cell parameters from 5426 reflections
*b* = 11.7210 (14) Å	θ = 2.2–27.4°
*c* = 11.9640 (14) Å	µ = 1.81 mm^−^^1^
α = 82.814 (1)°	*T* = 293 K
β = 88.854 (1)°	Block, black
γ = 76.644 (1)°	0.22 × 0.20 × 0.16 mm
*V* = 1268.5 (3) Å^3^	

### Data collection

**Table:**

Bruker SMART APEX CCD area-detector diffractometer	4692 independent reflections
Radiation source: sealed tube	4083 reflections with *I* > 2σ(*I*)
graphite	*R*_int_ = 0.028
phi and ω scans	θ_max_ = 25.5°, θ_min_ = 1.7°
Absorption correction: multi-scan (*SADABS*; Bruker, 2000)	*h* = −11→11
*T*_min_ = 0.692, *T*_max_ = 0.761	*k* = −14→14
9520 measured reflections	*l* = −14→14

### Refinement

**Table:**

Refinement on *F*^2^	Secondary atom site location: difference Fourier map
Least-squares matrix: full	Hydrogen site location: inferred from neighbouring sites
*R*[*F*^2^ > 2σ(*F*^2^)] = 0.030	H-atom parameters constrained
*wR*(*F*^2^) = 0.082	*w* = 1/[σ^2^(*F*_o_^2^) + (0.037*P*)^2^ + 0.5172*P*] where *P* = (*F*_o_^2^ + 2*F*_c_^2^)/3
*S* = 1.04	(Δ/σ)_max_ < 0.001
4692 reflections	Δρ_max_ = 0.38 e Å^−^^3^
290 parameters	Δρ_min_ = −0.34 e Å^−^^3^
0 restraints	Extinction correction: *SHELXL97* (Sheldrick, 2008), Fc^*^=kFc[1+0.001xFc^2^λ^3^/sin(2θ)]^-1/4^
Primary atom site location: structure-invariant direct methods	Extinction coefficient: 0.0118 (8)

### Special details

**Table:**

Geometry. All e.s.d.'s (except the e.s.d. in the dihedral angle between two l.s. planes) are estimated using the full covariance matrix. The cell e.s.d.'s are taken into account individually in the estimation of e.s.d.'s in distances, angles and torsion angles; correlations between e.s.d.'s in cell parameters are only used when they are defined by crystal symmetry. An approximate (isotropic) treatment of cell e.s.d.'s is used for estimating e.s.d.'s involving l.s. planes.
Refinement. Refinement of *F*^2^ against ALL reflections. The weighted *R*-factor *wR* and goodness of fit *S* are based on *F*^2^, conventional *R*-factors *R* are based on *F*, with *F* set to zero for negative *F*^2^. The threshold expression of *F*^2^ > σ(*F*^2^) is used only for calculating *R*-factors(gt) *etc*. and is not relevant to the choice of reflections for refinement. *R*-factors based on *F*^2^ are statistically about twice as large as those based on *F*, and *R*- factors based on ALL data will be even larger.

### Fractional atomic coordinates and isotropic or equivalent isotropic displacement parameters (Å^2^)

**Table:**

	*x*	*y*	*z*	*U*_iso_*/*U*_eq_	
Ni1	0.17029 (3)	0.03411 (2)	0.11584 (2)	0.03211 (11)	
S1	0.32210 (7)	−0.05513 (5)	−0.00222 (5)	0.03971 (16)	
S2	0.05550 (7)	−0.10606 (5)	0.14274 (5)	0.04056 (16)	
S3	0.09306 (7)	−0.33353 (5)	0.04221 (5)	0.03969 (16)	
S4	0.34360 (8)	−0.29296 (6)	−0.08879 (6)	0.04871 (18)	
S5	0.24451 (11)	−0.51768 (7)	−0.09332 (7)	0.0698 (3)	
S6	0.28357 (7)	0.17691 (5)	0.09083 (6)	0.03971 (16)	
S7	0.01554 (7)	0.11932 (5)	0.23353 (6)	0.04310 (17)	
S8	−0.00090 (8)	0.34602 (6)	0.33578 (6)	0.04481 (17)	
S9	0.23172 (7)	0.40488 (5)	0.19675 (6)	0.03994 (16)	
S10	0.09387 (8)	0.56899 (6)	0.35872 (6)	0.04947 (19)	
C1	0.2605 (3)	−0.1815 (2)	−0.00797 (19)	0.0345 (5)	
C2	0.1445 (3)	−0.20223 (19)	0.05421 (19)	0.0325 (5)	
C3	0.2283 (3)	−0.3877 (2)	−0.0501 (2)	0.0436 (6)	
C4	0.1888 (3)	0.27107 (19)	0.1800 (2)	0.0333 (5)	
C5	0.0760 (3)	0.2457 (2)	0.2430 (2)	0.0355 (5)	
C6	0.1081 (3)	0.4452 (2)	0.3007 (2)	0.0363 (5)	
C7	0.6079 (3)	0.9937 (2)	0.3192 (2)	0.0501 (7)	
H7	0.5323	1.0062	0.2667	0.060*	
C8	0.6073 (3)	1.0755 (2)	0.3933 (2)	0.0485 (6)	
C9	0.7202 (3)	1.0565 (2)	0.4706 (2)	0.0498 (7)	
C10	0.8302 (3)	0.9562 (3)	0.4754 (3)	0.0578 (8)	
H10	0.9047	0.9426	0.5290	0.069*	
C11	0.8304 (3)	0.8754 (2)	0.4010 (2)	0.0512 (7)	
H11	0.9062	0.8080	0.4036	0.061*	
C12	0.7197 (3)	0.8940 (2)	0.3231 (2)	0.0445 (6)	
C13	0.7260 (4)	0.8061 (3)	0.2393 (2)	0.0553 (8)	
H13A	0.6883	0.8484	0.1671	0.066*	
H13B	0.8274	0.7663	0.2292	0.066*	
C14	0.6972 (3)	0.6255 (2)	0.3536 (2)	0.0453 (6)	
H14	0.7872	0.6219	0.3868	0.054*	
C15	0.6241 (3)	0.5380 (2)	0.3842 (2)	0.0483 (6)	
H15	0.6653	0.4742	0.4371	0.058*	
C16	0.4906 (3)	0.5447 (3)	0.3368 (2)	0.0522 (7)	
H16	0.4399	0.4858	0.3569	0.063*	
C17	0.4328 (3)	0.6394 (3)	0.2594 (3)	0.0576 (8)	
H17	0.3413	0.6460	0.2272	0.069*	
C18	0.5084 (3)	0.7237 (2)	0.2294 (2)	0.0532 (7)	
H18	0.4691	0.7873	0.1760	0.064*	
Cl1	0.72732 (13)	1.15957 (9)	0.56077 (8)	0.0904 (3)	
Cl2	0.46624 (11)	1.19991 (8)	0.38728 (9)	0.0858 (3)	
N1	0.6396 (2)	0.71609 (18)	0.27623 (16)	0.0396 (5)	

### Atomic displacement parameters (Å^2^)

**Table:**

	*U*^11^	*U*^22^	*U*^33^	*U*^12^	*U*^13^	*U*^23^
Ni1	0.03753 (19)	0.02357 (17)	0.03463 (18)	−0.00516 (12)	0.00213 (13)	−0.00512 (12)
S1	0.0444 (4)	0.0296 (3)	0.0457 (4)	−0.0097 (3)	0.0117 (3)	−0.0064 (3)
S2	0.0457 (4)	0.0316 (3)	0.0478 (4)	−0.0123 (3)	0.0151 (3)	−0.0138 (3)
S3	0.0490 (4)	0.0300 (3)	0.0430 (4)	−0.0122 (3)	0.0052 (3)	−0.0103 (3)
S4	0.0604 (4)	0.0377 (4)	0.0490 (4)	−0.0094 (3)	0.0197 (3)	−0.0155 (3)
S5	0.1137 (7)	0.0433 (4)	0.0602 (5)	−0.0236 (4)	0.0240 (5)	−0.0297 (4)
S6	0.0433 (4)	0.0307 (3)	0.0478 (4)	−0.0105 (3)	0.0124 (3)	−0.0134 (3)
S7	0.0522 (4)	0.0326 (3)	0.0487 (4)	−0.0155 (3)	0.0160 (3)	−0.0123 (3)
S8	0.0539 (4)	0.0346 (3)	0.0484 (4)	−0.0112 (3)	0.0161 (3)	−0.0153 (3)
S9	0.0424 (4)	0.0286 (3)	0.0513 (4)	−0.0096 (3)	0.0080 (3)	−0.0127 (3)
S10	0.0481 (4)	0.0387 (4)	0.0655 (5)	−0.0078 (3)	0.0055 (3)	−0.0265 (3)
C1	0.0414 (13)	0.0279 (11)	0.0322 (12)	−0.0032 (10)	0.0038 (10)	−0.0049 (9)
C2	0.0395 (13)	0.0240 (11)	0.0327 (12)	−0.0041 (9)	−0.0026 (10)	−0.0041 (9)
C3	0.0615 (17)	0.0322 (13)	0.0366 (13)	−0.0067 (12)	0.0040 (12)	−0.0107 (10)
C4	0.0379 (13)	0.0244 (11)	0.0369 (12)	−0.0051 (9)	−0.0010 (10)	−0.0045 (9)
C5	0.0416 (13)	0.0273 (11)	0.0365 (13)	−0.0036 (10)	0.0017 (10)	−0.0085 (10)
C6	0.0377 (13)	0.0286 (12)	0.0406 (13)	−0.0012 (10)	−0.0040 (10)	−0.0084 (10)
C7	0.0596 (17)	0.0500 (16)	0.0416 (15)	−0.0162 (14)	−0.0084 (13)	−0.0006 (12)
C8	0.0566 (17)	0.0423 (15)	0.0439 (15)	−0.0076 (13)	0.0006 (13)	−0.0019 (12)
C9	0.0634 (18)	0.0460 (15)	0.0425 (15)	−0.0130 (13)	0.0014 (13)	−0.0142 (12)
C10	0.0571 (18)	0.0591 (18)	0.0574 (18)	−0.0101 (14)	−0.0133 (14)	−0.0115 (15)
C11	0.0505 (16)	0.0456 (15)	0.0566 (17)	−0.0074 (13)	0.0037 (13)	−0.0099 (13)
C12	0.0595 (17)	0.0406 (14)	0.0381 (14)	−0.0205 (13)	0.0094 (12)	−0.0071 (11)
C13	0.083 (2)	0.0518 (16)	0.0410 (15)	−0.0342 (16)	0.0201 (14)	−0.0110 (12)
C14	0.0390 (14)	0.0527 (16)	0.0428 (15)	−0.0092 (12)	−0.0013 (11)	−0.0024 (12)
C15	0.0506 (16)	0.0469 (15)	0.0432 (15)	−0.0080 (12)	0.0037 (12)	0.0039 (12)
C16	0.0549 (17)	0.0564 (17)	0.0527 (17)	−0.0257 (14)	0.0091 (14)	−0.0125 (14)
C17	0.0458 (16)	0.069 (2)	0.0609 (19)	−0.0165 (15)	−0.0098 (14)	−0.0116 (16)
C18	0.0634 (19)	0.0434 (15)	0.0472 (16)	−0.0029 (13)	−0.0158 (14)	0.0007 (12)
Cl1	0.1180 (8)	0.0762 (6)	0.0815 (6)	−0.0117 (5)	−0.0170 (6)	−0.0447 (5)
Cl2	0.0858 (6)	0.0596 (5)	0.0972 (7)	0.0171 (5)	−0.0164 (5)	−0.0142 (5)
N1	0.0488 (12)	0.0408 (12)	0.0321 (11)	−0.0139 (10)	0.0058 (9)	−0.0106 (9)

### Geometric parameters (Å, °)

**Table:**

Ni1—S2	2.1518 (7)	C8—C9	1.380 (4)
Ni1—S7	2.1643 (7)	C8—Cl2	1.722 (3)
Ni1—S6	2.1681 (7)	C9—C10	1.369 (4)
Ni1—S1	2.1714 (7)	C9—Cl1	1.731 (3)
S1—C1	1.719 (2)	C10—C11	1.378 (4)
S2—C2	1.708 (2)	C10—H10	0.9300
S3—C3	1.725 (3)	C11—C12	1.370 (4)
S3—C2	1.739 (2)	C11—H11	0.9300
S4—C3	1.738 (3)	C12—C13	1.515 (4)
S4—C1	1.750 (2)	C13—N1	1.493 (3)
S5—C3	1.642 (2)	C13—H13A	0.9700
S6—C4	1.717 (2)	C13—H13B	0.9700
S7—C5	1.721 (2)	C14—N1	1.336 (3)
S8—C6	1.727 (2)	C14—C15	1.369 (4)
S8—C5	1.742 (2)	C14—H14	0.9300
S9—C6	1.717 (3)	C15—C16	1.365 (4)
S9—C4	1.742 (2)	C15—H15	0.9300
S10—C6	1.662 (2)	C16—C17	1.368 (4)
C1—C2	1.356 (3)	C16—H16	0.9300
C4—C5	1.353 (3)	C17—C18	1.353 (4)
C7—C12	1.374 (4)	C17—H17	0.9300
C7—C8	1.383 (4)	C18—N1	1.340 (3)
C7—H7	0.9300	C18—H18	0.9300
			
S2—Ni1—S7	85.25 (3)	C7—C8—Cl2	119.6 (2)
S2—Ni1—S6	179.02 (3)	C10—C9—C8	120.1 (3)
S7—Ni1—S6	93.77 (2)	C10—C9—Cl1	119.0 (2)
S2—Ni1—S1	93.18 (3)	C8—C9—Cl1	120.9 (2)
S7—Ni1—S1	178.41 (3)	C9—C10—C11	120.0 (3)
S6—Ni1—S1	87.80 (3)	C9—C10—H10	120.0
C1—S1—Ni1	101.80 (8)	C11—C10—H10	120.0
C2—S2—Ni1	102.21 (8)	C12—C11—C10	120.3 (3)
C3—S3—C2	97.20 (12)	C12—C11—H11	119.8
C3—S4—C1	97.08 (12)	C10—C11—H11	119.8
C4—S6—Ni1	101.19 (8)	C11—C12—C7	119.8 (2)
C5—S7—Ni1	101.47 (9)	C11—C12—C13	119.1 (3)
C6—S8—C5	97.19 (11)	C7—C12—C13	121.0 (3)
C6—S9—C4	97.89 (11)	N1—C13—C12	112.5 (2)
C2—C1—S1	120.90 (18)	N1—C13—H13A	109.1
C2—C1—S4	115.57 (18)	C12—C13—H13A	109.1
S1—C1—S4	123.50 (14)	N1—C13—H13B	109.1
C1—C2—S2	121.87 (17)	C12—C13—H13B	109.1
C1—C2—S3	116.76 (18)	H13A—C13—H13B	107.8
S2—C2—S3	121.35 (14)	N1—C14—C15	120.3 (2)
S5—C3—S3	121.91 (17)	N1—C14—H14	119.9
S5—C3—S4	124.71 (17)	C15—C14—H14	119.9
S3—C3—S4	113.37 (13)	C16—C15—C14	119.7 (3)
C5—C4—S6	122.13 (17)	C16—C15—H15	120.1
C5—C4—S9	115.30 (17)	C14—C15—H15	120.1
S6—C4—S9	122.56 (14)	C15—C16—C17	118.9 (3)
C4—C5—S7	121.35 (18)	C15—C16—H16	120.6
C4—C5—S8	116.38 (17)	C17—C16—H16	120.6
S7—C5—S8	122.25 (15)	C18—C17—C16	120.1 (3)
S10—C6—S9	122.86 (15)	C18—C17—H17	119.9
S10—C6—S8	124.01 (15)	C16—C17—H17	119.9
S9—C6—S8	113.12 (13)	N1—C18—C17	120.5 (3)
C12—C7—C8	120.3 (3)	N1—C18—H18	119.7
C12—C7—H7	119.9	C17—C18—H18	119.7
C8—C7—H7	119.9	C14—N1—C18	120.5 (2)
C9—C8—C7	119.5 (3)	C14—N1—C13	119.1 (2)
C9—C8—Cl2	120.9 (2)	C18—N1—C13	120.4 (2)
			
S2—Ni1—S1—C1	1.40 (9)	Ni1—S7—C5—S8	−175.28 (13)
S6—Ni1—S1—C1	−178.64 (8)	C6—S8—C5—C4	1.5 (2)
S7—Ni1—S2—C2	177.99 (8)	C6—S8—C5—S7	−179.86 (15)
S1—Ni1—S2—C2	−1.80 (8)	C4—S9—C6—S10	178.91 (15)
S7—Ni1—S6—C4	1.33 (8)	C4—S9—C6—S8	−2.37 (15)
S1—Ni1—S6—C4	−178.88 (8)	C5—S8—C6—S10	179.61 (16)
S2—Ni1—S7—C5	177.64 (9)	C5—S8—C6—S9	0.91 (15)
S6—Ni1—S7—C5	−2.31 (9)	C12—C7—C8—C9	0.4 (4)
Ni1—S1—C1—C2	−0.6 (2)	C12—C7—C8—Cl2	−180.0 (2)
Ni1—S1—C1—S4	−178.50 (13)	C7—C8—C9—C10	−1.4 (4)
C3—S4—C1—C2	0.5 (2)	Cl2—C8—C9—C10	178.9 (2)
C3—S4—C1—S1	178.56 (16)	C7—C8—C9—Cl1	177.3 (2)
S1—C1—C2—S2	−1.1 (3)	Cl2—C8—C9—Cl1	−2.3 (4)
S4—C1—C2—S2	177.06 (12)	C8—C9—C10—C11	1.7 (5)
S1—C1—C2—S3	−179.55 (12)	Cl1—C9—C10—C11	−177.0 (2)
S4—C1—C2—S3	−1.4 (3)	C9—C10—C11—C12	−1.1 (5)
Ni1—S2—C2—C1	2.0 (2)	C10—C11—C12—C7	0.0 (4)
Ni1—S2—C2—S3	−179.53 (11)	C10—C11—C12—C13	177.6 (3)
C3—S3—C2—C1	1.6 (2)	C8—C7—C12—C11	0.3 (4)
C3—S3—C2—S2	−176.89 (15)	C8—C7—C12—C13	−177.2 (2)
C2—S3—C3—S5	177.82 (17)	C11—C12—C13—N1	95.9 (3)
C2—S3—C3—S4	−1.20 (17)	C7—C12—C13—N1	−86.5 (3)
C1—S4—C3—S5	−178.40 (18)	N1—C14—C15—C16	−1.2 (4)
C1—S4—C3—S3	0.59 (17)	C14—C15—C16—C17	0.0 (4)
Ni1—S6—C4—C5	0.4 (2)	C15—C16—C17—C18	1.0 (5)
Ni1—S6—C4—S9	179.73 (12)	C16—C17—C18—N1	−0.9 (5)
C6—S9—C4—C5	3.5 (2)	C15—C14—N1—C18	1.4 (4)
C6—S9—C4—S6	−175.90 (15)	C15—C14—N1—C13	−176.2 (2)
S6—C4—C5—S7	−2.6 (3)	C17—C18—N1—C14	−0.4 (4)
S9—C4—C5—S7	177.97 (12)	C17—C18—N1—C13	177.2 (3)
S6—C4—C5—S8	176.01 (13)	C12—C13—N1—C14	−78.8 (3)
S9—C4—C5—S8	−3.4 (3)	C12—C13—N1—C18	103.7 (3)
Ni1—S7—C5—C4	3.3 (2)		

### Hydrogen-bond geometry (Å, °)

**Table:**

*D*—H···*A*	*D*—H	H···*A*	*D*···*A*	*D*—H···*A*
C14—H14···S10^i^	0.93	2.82	3.622 (3)	145
C18—H18···S1^ii^	0.93	2.79	3.708 (3)	168

Symmetry codes: (i) *x*+1, *y*, *z*; (ii) *x*, *y*+1, *z*.
